# Valproic Acid-Induced Hyperammonemia in the Elderly: A Review of the Literature

**DOI:** 10.1155/2009/802121

**Published:** 2009-08-19

**Authors:** Vikrant Mittal, Sunanda Muralee, Rajesh R. Tampi

**Affiliations:** ^1^Parkland Health Center, 1103 West Liberty Street, Farmington, MO 63640, USA; ^2^Yale New Haven Psychiatric Hospital, 184 Liberty Street, New Haven, CT 06519, USA

## Abstract

Valproic acid and its derivatives are commonly used to treat many psychiatric conditions in the elderly. Hyperammonemia is a less common but important side effect of these drugs. The elderly patient appears highly vulnerable to this side effect of this group of medications. In this paper, we systematically review the published literature for hyperammonemia induced by valproic acid and its derivatives. We describe the three reported cases and review possible treatment strategies for this condition.

## 1. Introduction

Valproic acid is a broad-spectrum antiepileptic drug that is approved for the treatment of several types of seizures. It is also prescribed for the treatment of bipolar disorder, schizoaffective disorder, social phobias, neuropathic pain, and for the prophylaxis and treatment of migraine headaches [1]. Valproic acid and its derivatives have been found to be useful and generally safe in the treatment of bipolar affective disorder, agitation in dementia, and refractory anxiety disorders in the elderly [2–4]. These drugs are known to cause hyperammonemia and associated encephalopathy, but most of this data comes from children and younger adults [5–10]. However, recent case reports have highlighted the fact that this complication can also occur in elderly [4, 11, 12]. In this paper, we review the published literature on the occurrence of valproic acid-induced hyperammonemia in the elderly. 

## 2. Ammonia, Hyperammonemia, and Hyperammonemic Encephalopathy

Amino acids, products of protein breakdown, are usually metabolized in the liver by transaminases and oxidative deaminases to produce ammonia [13]. Ammonia is then converted to urea and excreted through the kidney. The most important enzyme in the incorporation of ammonia into the urea cycle is carbomylphosphatase synthetase I (CPS I) [14]. Ammonia is also produced in the kidneys by the conversion of glutamine to glutamate by the enzyme glutaminase [11]. 

 Hyperammonemia is a condition characterized by elevation in the serum level of ammonia above 40 mmols/L [15]. It is commonly caused by liver disease or inborn errors of metabolism [16]. Other less common causes are hyperinsulinemic hypoglycemia, carnitine deficiency, malignancies like leukaemias and myelomas, portosystemic shunts, urinary infections, surgeries, and parenteral nutrition [12, 13, 17–20]. Medications like 5-fluorouracil, salicylate, asparaginase, acetazolamide, diuretics, and valproic acid and its derivates have also been implicated in the development of this condition [5, 21–24]. However, an elevation in serum ammonia level above 200 mmols/L is more commonly seen in inborn errors of metabolism like urea cycle defects and branch chain organic acidaemias, although rarely other conditions may cause such high blood levels [25]. Although, hyperammonemia is thought to be associated with liver disease, most cases present with normal liver function [10, 26]. 

Mild and transient hyperammonemia can often be asymptomatic and is usually triggered by protein loads and catabolic states [13]. In symptomatic cases, the clinical features may be variable and episodic [13]. Many cases present with acute mental status changes characterized by confusion, personality change, irritability, ataxia, visual disturbance, lethargy, and somnolence [13]. They may also present with nausea, vomiting, or hyperventilation. More severe cases can lead to encephalopathy characterized by stupor and coma [27–30]. Although, most patients recover even from multiple episodes of hyperammonemic encephalopathy, it may occasionally lead to brain damage [31–33]. In severe untreated cases, seizures and death can occur due to raised intracranial pressure [13].

The pathogenesis of hyperammonemic encephalopathy remains unclear. Changes in mental status are thought to be due to high levels of ammonia and the presence of other organic acids. The brain ammonia concentration can be high even when the serum ammonia level is normal [5]. Hyperammonemia can also cause encephalopathy by inhibition of glutamate uptake by astrocytes [14, 34, 35]. As with delirium, elderly patients may present with several concomitant predisposing factors for developing hyperammonemia and encephalopathy [4, 36]. These include age-related cerebral changes like a more permeable blood brain barrier, alterations in stress-regulating neurotransmitters, and intracellular signal transduction systems [4, 36]. Other risk factors include multiple medical problems, such as renal failure or hypoalbuminemia, or concomitant administration of topiramate, cimetidine, and aspirin, that may elevate the free fraction of valproate and increase the risk of hyperammonemia even in the absence of metabolic defect in the urea cycle [4]. With age, protein binding and certain liver functions decrease, leading to decreased plasma albumin, hepatic oxidation, and hepatic cytochromes such as P-450-3A4 [4]. Concomitant administration of antiseizure medications such as phenytoin and phenobarbital may also increase the risk of hyperammonemia and should be used with caution [4]. In order to reduce the morbidity and mortality from this condition, vigilance for risk factors, early diagnosis, and prompt treatment are paramount [14].

It has been found that routine laboratory investigations are often not helpful in the diagnosis [13]. Although higher ammonia levels are associated with encephalopathy, blood levels of ammonia do not often correlate with the clinical condition [37]. It is important to ensure that serum ammonia levels are accurately measured as faulty storage and transport can lead to hemolysis and erroneous elevations in serum ammonia level [13]. Heparinised venous samples should be obtained and specimens should promptly be sent to the laboratory on ice or in a vacuum tube [13]. 

In patients with encephalopathy, cerebrospinal fluid (CSF) glutamine levels and EEG may aid in the diagnosis. A study of seven patients, ages 17–58 years, with valproate induced hyperammonemic encephalopathy (VHE), showed that elevation of CSF glutamine and the changes in the EEG correlated moderately well with the time course of the encephalopathy [14]. All patients had medically refractory seizures. Five patients had been initiated on valproate therapy shortly before developing signs and symptoms of VHE. Three of these patients had previously taken valproate without substantial adverse effects and two had never before received valproate. Two other patients had taken valproate for an extended period, but received a dosage increase before the onset of VHE. Dose of valproate ranged from 500 mg/day to 6500 mg/day, although the doses of valproate for 2 of the cases were uncertain. The valproate levels varied from 33–199 mg/dL and the ammonia levels were between 19–750 mmols/L). All patients in the study had EEG changes showing slow background waves with or without triphasic waves. CSF glutamine level was elevated in 80% of the patients with only 71% of patients having an elevated venous ammonia level. CT scan or MRI may aid in the diagnosis of encephalopathy [38]. These studies may reveal cerebral edema, although this is not a very consistent finding [39–41].

## 3. Methods

We searched the following databases: Ovid MEDLINE (http://gateway.tx.ovid.com/gw2/ovidweb.cgi) from 1950 to June Week 3 2009, EBM Reviews—Cochrane Database of Systematic Reviews 2nd Quarter 2009, PsycINFO 1967 to June Week 3 2009 with keywords “divalproex sodium,” “valproate,” “valproic acid,” and “divalproate,” “hyperammonemia,” “hepatic encephalopathy,” “urea cycle disorder,” “liver failure,” “aged,” and “elderly.” For the purpose of this paper, we considered anyone over the age of 60 years as “aged” or “elderly.” We only considered reports from peer reviewed English language journals for this review. For standardization purpose, we used micromols/liter (mmols/L) for ammonia level and milligrams/deciliter (mg/dL) for valproic acid level. Also, the terms “valproic acid” and “valproate” have been used synonymously.

## 4. Results

Most of the reported cases of valproate induced hyperammonemia are in children and younger adults. To date, there are only four reported cases of hyperammonemia in the elderly in the medical literature [4, 11, 12, 42]. Three major trials using valproic acid and its derivatives for the treatment of behavioral disturbance in dementia did not report hyperammonemia as a side effect. In these trials, sedation and urinary tract infection occurred more frequently in patients treated with valproic acid than in controls. Due to the differences in identifying adverse effects it was not possible to pool other observations concerning adverse effects between these studies [42].

The first reported case involved an 88-year-old African American man who presented with confusion after being treated with valproate at 250 mg qid for seizure disorder of unknown etiology [4]. He had high ammonia levels (836 mmols/L) despite normal liver function tests and valproate level (48 mg/dL). The confusion resolved once that the valproate was discontinued and phenytoin was started. The confusion returned once that the valproate was reinitiated at 250 mg po qid. The ammonia level during the second trial was 130 mmols/L and it decreased to 60 mmols/L once the valproate was discontinued.

McCall and Bourgeois reported a case in a 62-year-old person with brain aneurysm repair resulting in seizure disorder and being treated with divalproex sodium 250 mg tid [12]. The patient presented with a one-week history of confusion, mumbling speech, dizziness, lethargy, and weakness. The valproate levels were within normal range at 75 mg/L. The CT scan revealed increased size of the ventricles, sulci, and cisternal spaces with volume loss—mildly prominent for age. Ammonia levels were elevated at 99 mmols/L. The urinalysis and blood tests were within normal levels. The next day, the valproate was stopped and within next 3 days the patient's mental status improved. No other medication was used to reduce the ammonia level.

The third case involved a 76-year-old white woman with a seizure disorder who was taking divalproex sodium at 750 mg po tid [11]. She presented to the emergency room two months after treatment was initiated with divalproex, with a one week history of generalized weakness, confusion, nausea, and vomiting. She had normal liver function tests. The discontinuation of the divalproex sodium reduced the ammonia level from 211 mmol/L to 34 mmol/L within 11 days. The patient's mental status and physical functioning improved significantly after the divalproex was discontinued. She was then treated with gabapentin at 300 mg po bid for her seizure disorder.

The fourth case involved a 76-year-old man with bipolar illness who had been successfully treated with divalproex for the past 11 years and with extended-release divalproex for the past 16 months. He had consistently done well, serum valproic acid level was generally in the 50 to 80 mg/dL range, and transaminases were never elevated. There was no evidence of delirium or other problems attributable to divalproex; there had been 1 normal serum ammonia level 4 years ago. He became acutely confused, lethargic, and near-mute; he also began to urinate on the floor and to repeatedly remove his prosthetic eye. He was admitted with a serum ammonia level of 214 mmols/L. Serum valproic acid level was 73 mg/dL, and transaminases and liver ultrasound were normal. No other source of delirium was found. Divalproex was discontinued and he was treated with lactulose, the delirium cleared, serum ammonia normalized within 72 hours, and he was discharged. The author indicates that the reasons for this patient's encephalopathy remain unclear as there were no medication changes, no divalproex dosage adjustments, no evidence of liver disease, and no dietary change. This report underscores the fact that hyperammonemic encephalopathy is not necessarily an early complication of valproate therapy [42].

## 5. Discussion

The risk factors for the development of valproic acid-induced hyperammonemia include higher doses of the medication, concomitant use of other antilepiliptics like topiramate, carnitine deficiency, and the presence of congenital abnormalities of urea cycle [5, 12, 22, 24, 43]. The male to female ratio appears to be equal, although conclusive data is lacking at this time [44]. 

The mechanism by which valproic acid and its derivatives produce hyperammonemia is unclear, although various mechanisms have been proposed [45]. In the kidney, these drugs can increase the transport of glutamine across the mitochondrial membrane, thus making it available for the production of ammonia [46]. Furthermore, valproic acid can stimulate the enzyme glutaminase to increase the conversion of glutamine to glutamate, thereby making it more available for the production of ammonia [24, 45, 47, 48].

Valproic acid and its derivatives are also thought to inhibit the enzyme, Carbamoylphosphate synthetase I (CPS I), thereby preventing the incorporation of ammonia into the urea cycle [4, 5, 24, 30, 45, 49]. These drugs may inhibits N-acetylglutamate, an activator of CPS I, that is, needed for the normal function of this enzyme [50, 51]. They can increase the concentration of pyruvate, a potent inhibitor of CPS I which prevents the incorporation of ammonia into the urea cycle [3, 4, 24, 30, 45, 49].

Carnitine an important nutrient found in meat and dairy products is also essential for the metabolism of valproic acid [52]. Long-term treatment with valproic acid and its derivatives depletes the stores of carnitine, thus reducing the metabolism of the drug and increasing the risk for hyperammonemia [45, 53]. This condition is more common in children less than 2 years of age, those with inborn errors of metabolism and those patients on a vegetarian diet [22].

In patients with hyperammonemia, valproate levels may often be normal and the symptoms do not necessarily correlate with the degree of hyperammonemia, so clinical correlation is paramount [24, 54, 55]. L-carnitine supplementation has been shown to improve the symptoms of valproate related toxicities and reduces mortality in patients with valproate induced hepatotoxicity [56–58]. The use of intravenous hydration, 60 gm protein restriction, and lactulose 45 mL every 24 hours, in addition to stopping the valproate has been shown to reduce valproic acid-induced hepatotoxicity [58]. Monitoring of symptomatic patients remains clinical rather than based on the serum ammonia levels [12]. The primary treatment for most patients with valproic acid-induced hyperammonemia is the dose reduction or withdrawal of the offending drug, in which case, complete recovery often occurs within a few days [4, 56]. Addition of L-carnitine supplementation to dose reduction strategies may aid in faster resolution of symptoms [8, 52].

## 6. Conclusion

In elderly patients, valproic acid-induced hyperammonemia assumes significance, as many of these patients have comorbid conditions that already decrease their quality of life [59]. With the growing use of valproic acid and other mood stabilizers in the management of psychiatric conditions in the elderly, it has become important for clinicians to be aware of more uncommon adverse effects of valproate.

A systematic review of literature indicates that there are four case reports documenting the development of hyperammonemia due to valproic acid and its derivatives in the elderly. Three of the four cases developed when valproate was used in the treatment of seizure disorder. The fourth case occurred in an elderly man treated successfully for bipolar illness with divalproex. As the mechanism of valproate induced hyperammonemia is multifactorial, it is important to be vigilant when using this medication in the elderly patients who have many comorbid conditions and concurrently use multiple drugs.

Although the followup of the blood ammonia levels seems most intuitive in diagnosing hyperammonemia, often patients with elevated ammonia levels are asymptomatic [24, 54]. The data to recommend routine checking of ammonia levels in all elderly patients treated with valproate is unavailable. However, available data indicates that ammonia levels should be checked and closely monitored in all elderly patients treated with valproate who develop altered mental status regardless of the duration of therapy and especially during periods of acute illness [42]. The efficacy of EEG and imaging techniques like brain CT and MRI scans in the diagnosis of symptomatic patients remains inconclusive [14, 38].

From the review of literature it appears that the best treatment measure for hyperammonemia from valproic acid and its derivatives is the dose reduction or discontinuation of the drug with or without supplementation of L-carnitine and providing symptomatic treatment for the patient. In the meantime, we should continue to carefully followup elderly patients treated with valproate and its derivatives for the development of sides effects like hyperammonemia.
